# Holistic Patterns as an Instrument for Predicting the Performance of Promising Young Soccer Players – A 3-Years Longitudinal Study

**DOI:** 10.3389/fpsyg.2016.01088

**Published:** 2016-07-27

**Authors:** Claudia Zuber, Marc Zibung, Achim Conzelmann

**Affiliations:** Institute of Sport Science, University of BernBern, Switzerland

**Keywords:** person-oriented approach, pattern analysis, prediction, success, soccer

## Abstract

Multidimensional and dynamic talent models represent the current state of the art, but these demands have hardly ever been implemented so far. One reason for this could be the methodological problems associated with these requirements. This paper will present a proposal for dealing with this, namely for examining the development of young soccer players holistically. The patterns formed by the constructs net hope, motor abilities, technical skills and biological maturity were examined, as well as the way in which these holistic patterns are related to subsequent sporting success. 119 young elite soccer players were questioned and tested three times at intervals of 1 year, beginning at the age of 12. At the age of 15, the level of performance reached by the players was determined. At all three measuring points, four patterns were identified, which displayed partial structural and high individual stability. The *highly skilled players*, scoring above average on all factors – but not necessarily those having the highest overall scores – were significantly more likely to advance to the highest level of performance. *Failure-fearing fit players*, i.e., physically strong, early developed players but with some technical weaknesses, have good chances of reaching the middle performance level. In contrast, none of the *achievement-oriented, highly skilled, late-matured* or *late-matured, low skilled players* reached the highest performance level. The results indicate the importance of holistic approaches for predicting performance among promising soccer talents in the medium-term and thus provide valuable clues for their selection and promotion.

## Introduction

For some time now, there has been a call within talent research to use multidimensional and dynamic approaches to identify promising athletes at a young age and with a high probability of success (Williams and Franks, 1998; Abbott and Collins, 2002; Abbott et al., 2005; Reilly et al., 2008; Vaeyens et al., 2008; Meylan et al., 2010; Unnithan et al., 2012; Phillips et al., 2014). This means, on the one hand, that not only their current competitive performance should be used as a basis for the assessment, but that predictors should be considered that are drawn from various different endogenous (motor performance, psychological features, etc.) and exogenous (e.g., social support or characteristics of training) areas relevant to performance. On the other hand, the dynamic approach is meant to do justice to the development process of the athletes from a promising young talent through to a successful, top competitive athlete. This demand goes hand in hand with the assumption that top athletic performance is not only achieved via a single set of predictors, but rather through a multitude of different combinations of these properties, since a range of different compensatory effects, weightings, and rates of development are assumed to apply to the individual predictors. In other words, the athlete is meant to be conceptualized as “a complex, dynamical system” (Phillips et al., 2010, p. 272). One way of meeting these requirements, which is both theoretically founded and empirically implementable, is to use a person-oriented approach (Bergman and Magnusson, 1997; Bergman et al., 2003).

From a theoretical point of view, the appropriateness of the person-oriented approach for talent research can be justified as follows: since questions about talent development deal with human developmental processes, it is helpful to draw on current theories of human development. In developmental psychology and developmental science, dynamic interactionist approaches are favored when explaining human development (Magnusson, 1990; in sport science, Conzelmann, 2001). In addition to a dynamic interactionist perspective, which meets the demand for a dynamic understanding of talent, a holistic view of human development (Magnusson and Cairns, 1996) is also necessary. An individual functions and evolves as a holistic organism, whose various aspects do not develop independently of one another. The individual and his environment are regarded as a system (Magnusson and Stattin, 2006). Hence when analyzing human development, the individual should always be viewed as a whole. The person-environment system can be subdivided into different subsystems, which mutually interact with each other (Bergman and El-Khouri, 2003). This holistic approach leads to a change in perspective, from the – hitherto dominant – variable-oriented to a person-oriented approach. From an empirical point of view, the first implementations in talent research have proven to be promising (cf. Zibung and Conzelmann, 2013; Zuber et al., 2015).

The person-oriented approach (Bergman and Magnusson, 1997) has a number of methodological consequences. Firstly, the variables involved in a (sub)system need to be measured as completely as possible. Secondly, it is necessary to dispense with statistical methods based on the general linear model (GLM), since the reciprocal interactions between the variables mean that the assumption of linearity has to be sacrificed (Bergman and Magnusson, 1997). Pattern analyses are one possible method of implementing the person-oriented approach. In these, states of the system (so-called patterns) are depicted at different times and the transitions between these patterns are analyzed. The variables involved in a system are referred to here as operating factors (Bergman et al., 2003). For a more detailed overview of the person-oriented approach, cf. (Bergman et al., 2003), and for a comparison with the variable-oriented approach, cf. (Bergman and Andersson, 2010).

Due to the high complexity of the person-environment system, empirical studies using the person-oriented approach in talent research have so far often focused on single subsystems, such as motivation (Zuber et al., 2015) or training (Zibung and Conzelmann, 2013). The advantage in doing this is that the subsystems can be described in great detail. On the other hand, this procedure only partially satisfies the demand for a holistic approach. This incomplete implementation is justified with reference to research economy and the difficulty of measuring the entire person-environment system (cf. Bergman and Magnusson, 1997; Trost and El-Khouri, 2008; Zibung and Conzelmann, 2013). To approach a holistic concept more closely, it therefore makes sense to take into account operating factors from different areas relevant to performance instead of looking at a single subsystem.

In order to limit the variety of different factors for identifying talents, this paper will focus on endogenous properties. The starting point for selecting the areas to be considered is the multidimensional talent identification and development (TID) model proposed by Abbott et al. (2005). This is based on viewing talent as a complex, dynamic system that develops as it interacts with the key indicators. These “key performance determinants” are considered to be motor behaviors, psychological behaviors and physical characteristics, since they play an important role both in talent identification and in the process of talent development (Abbott et al., 2005, p. 61).

Looking at the current state of research, it is found that talent research in the field of soccer has until now only occasionally complied with the demand for multidimensional test batteries, in which features from two or more areas were included (e.g., Reilly et al., 2000b; Carling et al., 2009; Figueiredo et al., 2009; Le Gall et al., 2010; Huijgen et al., 2014). In most of these studies, the ability of individual variables to discriminate between different levels of performance was compared. Few studies using a holistic approach, in other words trying to portray athletes in their entirety, have been conducted so far. Only Huijgen et al. (2014) provide information about the ability of combinations of several properties to predict performance. They were able to classify 69% of talented players correctly using a combination of technical, tactical and physiological characteristics. As regards other team sports, one study is worth mentioning in particular. Using the mean rank of diverse motor and technical ability tests, as well as an evaluation of game intelligence, Falk et al. (2004) were able to correctly assign 67% of all water polo players either to the group of national team players or to the group of players who were not selected 2 years later.

Concerning the dynamic aspect, some of the studies mentioned include a follow-up assessing a performance criterion, e.g., continuation on the same or a higher level versus dropping out (Figueiredo et al., 2009), achieved status (professional versus amateur; Carling et al., 2009; Le Gall et al., 2010; Till et al., 2015) or selection versus deselection (Huijgen et al., 2014). Other studies did include several measuring points, but the stability of the test results or of the pattern of results was not determined and/or reported (Falk et al., 2004).

Clearly, therefore, more studies are needed that analyze talent development theoretically and empirically, both dynamically and holistically.

### The Present Research

In the light of this dearth of multidimensional and dynamic talent studies, the present longitudinal study among young soccer talents resorts to a person-oriented approach. Factors from the areas of motor behavior, psychological behaviors and physical characteristics are used to form patterns, which are subsequently used to identify developmental types that are particularly promising in adolescence in the medium term.

Technical skills and physical fitness are considered to be representative of the field of motor behavior. They are mentioned in papers on the profile of demands made in soccer (e.g., Reilly et al., 2008) and have also been shown to have predictive power in various different studies, or else they are at least able to distinguish between groups with different levels of performance (Reilly et al., 2000b; Russell et al., 2010; Gonaus and Müller, 2012; Huijgen et al., 2014; Höner et al., 2015). In the field of psychological behaviors, net hope – a combination of two components of the achievement motive – is chosen as an indicator of the achievement motive (Brunstein and Heckhausen, 2010), since motivational properties in the field of personality have been shown in previous studies to be relevant predictors (Coetzee et al., 2006; van Yperen, 2009; Feichtinger and Höner, 2014; Zuber and Conzelmann, 2014; Zuber et al., 2015). Biological maturity is chosen as the operating factor for the field of physical characteristics. It is confounded both with the current level of physical performance (Vandendriessche et al., 2012) and to some extent also with the technical skills (Malina et al., 2005a), and should therefore be taken into account as well when making selection decisions (for an overview: Meylan et al., 2010).

As combinations of features from different areas of performance have rarely been studied for predicting performance in soccer, no conclusions can be drawn about possible connections between global patterns and success in sports. However, the current state of research on the connection between individual variables and success in soccer indicates that a high expression of the operating variables net hope (Coetzee et al., 2006; Feichtinger and Höner, 2014; Zuber and Conzelmann, 2014; Zuber et al., 2015), technique and fitness (Reilly et al., 2000b; Russell et al., 2010; Gonaus and Müller, 2012; Huijgen et al., 2014; Höner et al., 2015) seems to be promising, at least cross-sectionally and in some cases also longitudinally. However, if the high degree of physical fitness and, to a lesser extent, of technique are linked to early maturation, then some caution is necessary in assessing them, since it may be assumed that this advantage in motor performance is perhaps largely due to biological maturity and therefore will not be stable in the long run (Le Gall et al., 2002).

The concept of stability and change is central to talent selection and promotion (Feichtinger and Höner, 2015). With regard to pattern-analytical methods of analysis, this means that after determining the patterns, their stability also needs to be examined. In the context of the person-orientated approach, two different types of stability are distinguished (cf. Bergman et al., 2003). Structural stability (SS) refers to the similarity of the patterns found at different measuring points. If the patterns are replicated in similar forms (i.e., similar cluster centroids are found), they are said to display high SS. If the patterns remain stable over time on a group level (SS), then it follows that one can look for the same patterns at different points in time. Individual stability occurs if certain developmental paths are particularly frequent on an individual level. If these developmental paths are in addition associated with success in sports, then promoting this type of player has a higher chance of leading to success. If individual stability occurs between structurally stable patterns, i.e., if a particularly large number of athletes follow the path between structurally similar clusters, it may in addition be assumed that the time at which a type is assigned does not make any difference, which is particularly valuable when it comes to talent selection.

As there are to our knowledge no studies using the person-oriented approach and taking into account different subsystems, no assumptions about the results can be formulated. Hence, the exploratory analysis will be guided by the following research questions:

(1) Which patterns can be identified in young talented soccer players, in terms of the four operating factors net hope, technical skills, physical fitness and maturity status?
(2) How stable are these patterns over two 1-year periods (SS)?
(3) What development paths do young talented soccer players follow during this time (individual stability)?
(4) Do patterns exist that coincide particularly closely with sports success a year on?

## Materials and Methods

### Participants and Procedure

Beginning in summer 2011 (*t*_1_), the members of six U13 regional teams of the Swiss Football Association were recruited for the study and took part in three tests, 1 year apart each. All in all, 172 young male soccer talents took part in at least one of the three tests. The data analysis only considered those players who were present for at least two of the three tests (*n* = 123; *M*_Age_ = 12.26, *SD* = 0.29). In addition, four outliers have been removed (see Residual Analysis), so that the final sample includes *n* = 119 players who were tested at the ages 12.27 ± 0.29, 13.33, and 14.33 years, at t_1_, t_2_, and t_3_, respectively. On these testing days, the operating variables were ascertained by means of questionnaires, sport-motor tests and anthropometric measurements. One year after t_3_, the performance level of the players at the age U16 was assessed. Talent cards which are allocated by the Swiss Football Association served as indicators. The best players (national team) receive national, talented players receive regional talent cards. Thus, three performance levels can be distinguished: players with a national (*n* = 12), a regional (*n* = 39), or no talent card (*n* = 68). Players holding a national talent card are mostly members of the U16 national team.

The study was approved by the ethics committee of the Philosophical-Humanistic Faculty at the University of Bern. All players and their parents or legal representatives gave their written informed consent to participate in the study.

### Measures

#### Net Hope

Net hope (NH) is measured as the difference between the two components of the achievement motive: hope for success (HS) and fear of failure (FF; NH = HS – FF; Brunstein and Heckhausen, 2010). These were measured using the German version of the short scale of the Achievement Motives Scale – Sport (AMS-Sport; Wenhold et al., 2009). Each scale consists of five items, with a four-point response scale (from 0 = ‘does not apply to me at all’ to 3 = ‘applies to me completely’). The internal consistencies were acceptable for group comparisons, with α_HS__t1/t2/t3_ = 0.72/0.76/0.82 and α_FF__t1/t2/t3_ = 0.77/0.76/0.83. Positive scores for net hope indicate that hope for success is greater than fear of failure.

#### Physical Fitness

The motor abilities were determined by means of three motor tests measuring speed (40-m sprint, *r*_tt_ = 0.96), intermittent endurance (Yo-yo IR1 Test; Bangsbo et al., 2008) and jumping strength (countermovement jump; Casartelli et al., 2010)^1^. To obtain the physical fitness score, the best score on each of these tests (only one attempt at the Yo-yo Test) was taken, *z*-standardized and combined with the other test scores to produce a mean. In doing so, the test results were aligned such that positive scores stand for above-average performance.

#### Technical Skills

Technical skills were captured by three tests assessing dribbling in a slalom course (*r*_tt_ = 0.52), ball control by passing against impact walls (*r*_tt_ = 0.68) and juggling by reciprocally changing legs (*r*_tt_ = 0.79)^2^. For a detailed description of the individual tests, see Höner et al. (2015). Each of these technical tests was carried out twice. To produce the technical skills dimensions, the best score for each test was selected, *z*-standardized and combined with the other test scores to produce a mean. In doing so, the test results were aligned such that positive scores stand for above-average performance.

#### Biological Maturity

Biological maturity was determined using the test put forward by Mirwald et al. (2002). This allows the adult height to be predicted using maturity-based cumulative height velocity curves. It is a non-invasive, simple and inexpensive method, in which only the exact measurements of weight, standing height and sitting height are needed. These anthropometric measurements were carried out twice each. When the deviation between the measurements was greater than 4 mm or 0.4 kg, the measurement was repeated. The measure used was the mean of the data collected. “As it is known that early maturing individuals…are closer to their adult height than average and late-maturing individuals of the same chronological age” (Sherar et al., 2005, p. 508), it can be deduced that the current percentage of the adult height is a valid estimate of biological maturity (Malina et al., 2005b).

### Data Analysis

#### Data Processing

The subsequent cluster analyses require complete sets of data, and since 63 of the 123 players only took part in two of the three measuring points, their missing values were imputed. Little’s MCAR test indicated a non-significant result (χ^2^ = 123.0, *df* = 103, *p* = 0.09), meaning that the missing data are completely random and an imputation by means of expectation maximization (EM) is legitimate (Tabachnick and Fidell, 2013). Overall, 18.43% of the data were imputed by EM^3^.

#### LICUR Method

The LICUR method (**Li**nking of **C**l**u**sters after removal of a **R**esidue, cf. Bergman et al., 2003) is a pattern-analytical procedure that is suitable for implementing person-oriented approaches. The fundamental idea behind it is to form clusters (patterns) within each developmental phase. In order to map the developmental process, the individual transitions are then determined, either from the clusters of one phase to those in the next phase, or to a specific developmental outcome. The LICUR method consists of three steps. First, a residual analysis is carried out, in which extreme cases (residues) are identified and removed from the data set, since they would distort the cluster solution. In the next step, clusters are formed for the specific phases (cluster analysis). In the final step, the similarity between the patterns of the different phases is determined (SS) and more especially the developmental (anti-)types are established (individual stability). The statistical methods applied in the first and second steps are based on the GLM; whereas in the third step, transition probabilities between patterns or developmental outcomes are determined. In other words, as suggested by the systemic development concepts, the development of the motivation types is not based on linear or continuous functions. The first and third steps were carried out using the statistics package SLEIPNER 2.1 (Bergman and El-Khouri, 2002), while the cluster analysis was done using SPSS Statistics 22.0.

##### Residual analysis

During residual analysis, the patterns of pairs of subjects are compared. Any subject not displaying similarities with at least a predetermined number of other subjects (squared Euclidian distance) is identified as being a residue. According to Bergman et al. (2003), the number of residues should not exceed 3% of the total sample size. For the analysis, a threshold value of *T* = 0.8 was chosen for the distance. The minimum number of similar cases was set to be *K* = 1, meaning that only those subjects were excluded whose pattern is unique. Particularly when studying talent development, such residues can provide important insights into the developmental process, since unique achievements may be the result of unique developmental paths. However, relevant insights can also be gained from the understanding characteristic properties of players who are not successful.

##### Cluster analysis

Ward’s method, using the squared Euclidian distance as a distance measure, was chosen for the cluster analysis (Everitt, 2011), as recommended in the literature for person-oriented approaches (Bergman et al., 2003; Trost and El-Khouri, 2008). The choice of the best cluster solution was guided by content (interpretability of the clusters) as well as statistical criteria. The statistical criteria included the elbow criterion, as well as the Mojena test (Everitt, 2011) with a threshold value of 2.75. Furthermore, solutions with the smallest number of an *F* scores >1 were favored (Backhaus et al., 2008). Afterward, the cluster solutions that had been found were then optimized by subjecting them to a cluster center analysis (Backhaus et al., 2008).

##### Structural stability

**FIGURE 1:**
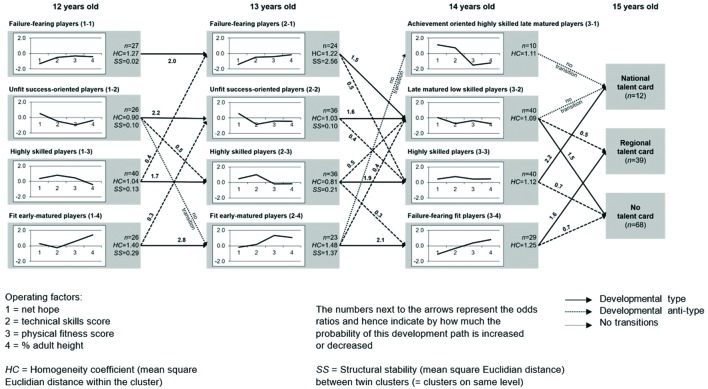
***z*-score profiles of the clusters (cluster centroids) and developmental (anti-)types for the ages 12, 13 and 14, and the performance criterion at age 15**.

Structural stability refers to the similarity of the patterns found at different measuring points. If the patterns are replicated in similar forms (i.e., similar cluster centroids are found), they are said to display high SS. In order to analyze the SS, the average square Euclidian distance between the clusters is compared. The clusters are arranged in pairs by increasing value, meaning that the clusters that are most similar to each other end up next to each other at the same level (cf. **Figure 1**).

##### Individual stability (developmental types)

In order to analyze the individual developmental paths, the transitions between the clusters of one phase and those of the next phase, or a specific developmental outcome, are counted and checked for significant deviations from random variations (*p* < 0.05) using the exact Fisher 4-field distribution test based on a hypergeometric distribution. The odds ratio indicates the degree to which the probability of this developmental path has increased (developmental types) or decreased (developmental anti-types).

## Results

### Residues

In the first step of the LICUR method, two residues were identified and removed both in the first (#44, #126) and in the second (#25, #37) phase, which lies below the limit of 3% of the total sample proposed by Bergman et al. (2003). No residues were identified at the third measuring point. In the present case, all four were assigned to the lowest performance level. A common features shared by all four residues is that the scores on two of the four operating factors were in some cases clearly below or around *z* = -1.5 and therefore very substantially below average.

### Clusters

In the following cluster analysis, the stated criteria suggested a four-cluster solution at all three measuring points. The final cluster solution after the cluster center analysis displays an explained error sum of squares of 44.4% at *t*_1_, 47.0% at *t*_2_, and 44.4% at *t*_3_.

**Table 1** shows the descriptive statistics of the operating factors before *z*-standardization and also before the motor tests are averaged to produce the two scores technical skills and physical fitness. In addition, a one-factor repeated measures analysis of variance is used to decide whether there are differences in the test scores at the three measuring points. It will be noted that the players improved over time on all the motor tests - as one would expect. It is also found that the net hope scores are for the most part positive, and that they do not change with age. The percentage of the estimated adult height achieved lies between 83.63 and 86.73% at the age of 12, climbing to 88.80–94.94% at the age of 14. The variation is largest at the age of 13 years.

**Table 1:** Descriptive statistics of the operating variables at the three measuring points and one-way repeated measures ANOVA.

*n* = 119	12 years		13 years		14 years		ANOVA	
	*M*	*SD*	*M*	*SD*	*M*	*SD*	*F*(2,117)	*p*
Net hope	1.86	0.81	1.81	0.83	1.94	0.72	1.13	0.33
Dribbling (sec.)	10.71	0.87	10.14	0.87	10.20	0.68	20.45	<0.001
Ball control (sec.)	19.66	3.54	17.87	3.56	15.78	1.44	55.39	<0.001
Juggling (pts.)	2.75	3.20	5.28	6.46	8.04	6.98	43.33	<0.001
40 m sprint (sec.)	6.64	0.33	6.43	0.30	6.21	0.44	69.63	<0.001
Yo-yo (m)	865.67	284.81	1087.57	387.43	1330.33	456.79	55.83	<0.001
CMJ (cm)	28.83	3.73	30.60	3.76	30.56	3.80	16.33	<0.001
% adult height	84.34	1.69	87.54	2.48	92.48	3.03	1415.69	<0.001

*Lower values in time-relevant tests (dribbling, ball control, 40 m sprint) are indicative for better performance. In all the other tests, higher values stand for better performances*.

To answer our first research question, the respective means of the clusters at every measurement point are presented in **Figure 1** as *z*-standardized scores. In naming the clusters, only those operating factors with *z*-scores >| 0.5| were included in assigning a name. Hence, for example, cluster 1-1 was named after the distinctly below-average operating factor Net Hope (*z* = -1.3) and the players assigned to it are described accordingly as *failure-fearing players*. However it should be noted that comparisons, and therefore the description of being above- or below-average, are only permissible within the overall sample, which already represents a highly selected group. In all three age groups, there is one cluster that is less promising than the others – in accordance with the assumptions of the current state of research (see The Present Research): *failure-fearing players* (clusters 1-1 and 2-1) and *late-matured low-skilled players* (3-2), whose profile are primarily confined to the negative range. The clusters of *unfit success-oriented players* (1-2) and *low-skilled, success-oriented players* (2-2) can also be said to be less promising, as their only positive scores are in the field of net hope. The other two clusters at the age of 12 (1-3, 1-4) and 13 (2-3, 2-4) display a combination of positive and negative values for their operating factors. The players in the cluster of *highly skilled players* (1-3, 2-3, 3-3) display above-average scores for net hope and technical skills at all three age levels, and at the ages of 12 and 13 years these players are still comparatively late developers.

### Developmental Types and Antitypes

Furthermore, **Figure 1** shows the developmental types and antitypes between the ages 12, 13, 14 and the performance groups at age 15 respectively, which brings us to our second and third research questions. As expected, the four developmental types observable between 12 and 13 years occur between similar, i.e., structurally stable, clusters. This means that the *highly skilled players* at the age of 12, for example, are significantly more likely to be found in the cluster of *highly skilled players* at the age of 13, too. Thus there is a higher-than-random probability between 1.7 < OR < 2.8 that all players will continue to be in the similar group a year later. The developmental antitypes occur between dissimilar clusters. Thus for example the probability (OR = 0.4) of *highly skilled player* at the age of 12 being found among the *failure-fearing players* a year later is significantly lower. This suggests that it is rare for substantial changes in the pattern to occur over this period. In addition, one path between highly dissimilar clusters (1-2 to 2-4) was identified along which no transitions occurred. Not one 12-years-old *unfit success-oriented players* was found among the *fit early matured players* at the age of 13.

Four developmental types were also found between the ages of 13 and 14. Thus players from the two clusters (2-1 and 2-2) make the transition to cluster 3-2 at an above-average rate, Furthermore, it will be seen that not a single transition took place from cluster 2-4 to cluster 3-1. In addition, this cluster of *achievement-oriented, highly skilled, late-matured players* has little similarity to the already identified clusters, which suggests that it is a newly emerging profile, and furthermore it contains only a small number of players, at *n* = 10. The developmental types from cluster 2-3 to 3-3 and from 2-4 to 3-4 are, as expected, found to lead to the most similar cluster at the later time.

The transition probabilities between the age of 14 years and the U16 talent cards are of special interest in terms of the fourth question asked in this article – one that is particularly relevant to talent development and selection. Based on the way in which the individual operating factors are associated with performance in sports (see The Present Research), *highly skilled players* (3-3) with values (slightly) above average on all operating factors might be assumed to produce a higher-than-random number of players selected for the U16 national team. By contrast, *late-matured low-skilled players* (3-2) with below-average scores for their technical skills, physical fitness and maturity status might be assumed to receive a national talent card less often than chance would predict.

Looking at the transition probabilities from age 14 to the performance criterion at age 15, both these conjectures are confirmed: one developmental type occurs from the cluster of the *highly skilled players* to the highest performance level. These players are in addition found more rarely than on average at the lowest performance level. In contrast to this, not a single player manages to make the transition from the *late-matured, low-skilled players* (3-2) and the *achievement-oriented highly skilled late-matured players* (3-1) into the highest performance level. A second developmental type shows that *failure-fearing fit players* are nominated more often for the regional performance level than chance would suggest.^4^ In summary, it may be stated that the pattern of the *highly skilled players* is individually and to some extent structurally stable, and is furthermore associated to a particularly high degree with success in soccer. A high structural and individual stability is also seen in *failure-fearing fit players*. Those physically strong, early developed players but with some technical weaknesses, have good chances of reaching the middle performance level. The players from the other two clusters (3-1 and 3-2) are distinctly less likely to be successful.

## Discussion

This study set out to fulfill the much cited demand for looking at talents and their development in a complex and dynamic way. This makes it one of the first studies that take into account both the dynamic and the holistic aspect. To do this, the development of young talented soccer players was studied over a period of 3 years according to the principle of holistic-dynamic interactionism. The four operating factors – net hope, technical skills, physical fitness, and biological maturation – representing broad, performance-relevant dimensions, were used to establish patterns (research question 1). In a further step, the structural and individual stability of these patterns was analyzed and particularly promising holistic patterns were sought (research questions 2 and 3).

In doing so, four clusters were identified, which were structurally stable over a period of 1 year between the age of 12 and 13 years. Together with the high individual stability between twin clusters at ages 12 and 13, this result suggests that the holistic system used is relatively stable over this time period.

Between the age of 13 and 14 years, SS decreased a bit. As a new cluster was formed, the cluster of the *achievement-oriented highly skilled late-matured players* we can speak of partial SS (Bergman et al., 2003). The transition from the age of 13 to 14 (individual stability) is characterized by an increased level of change in two (2-1, 2-2) of the four clusters identified. The other two clusters, namely the *highly skilled players* and the *fit early matured players*, display a (relatively) high SS, however, and the players display a high individual stability.

Referring to the fourth research question, these two types of players also appear to be the most promising ones: *failure-fearing fit players*, i.e., physically strong, precociously developed players, though having some technical and motivational weaknesses, have good chances of reaching the middle performance level. This level of performance is only achieved by 1% of all licensed soccer players in a particular age group in Switzerland. This comparatively high probability of success in early developing soccer players matches previous findings (for an overview, cf. Malina et al., 2013), from variable-oriented studies. The *highly skilled players* are still among the late developers at the age of 12 and 13, however, they appear to be able to make up for this deficit over the next year and then have more than twice as good a chance of achieving the top level of performance, which includes only 0.3% of all 15-years-old soccer players in Switzerland. It therefore appears as though delayed development at the age of 12 and 13 is compensated by a high level of technical skills and net hope. Players who are still late-developers at the age of 14, however, and who may for this reason have weaknesses in their physical fitness (cf. clusters 3-1 and 3-2), do not display any above-random frequency of transition to high-performance groups. On the contrary, none of the players in these two clusters are found to have made the transition to the top level of performance. This does to rule out, however, that they might still make the transition at a later time (Ostojic et al., 2014).

As is generally expected in talent development (Meylan et al., 2010), additional compensation effects are also observed in the holistic clusters found in this study: below-average technical skills and low net hope can be at least partially compensated during the year before the selection, leading to a higher probability of transition into the intermediate, though not into the top level of performance. However, it appears that extremely negative scores on half the operating factors, as observed in the residues and in the *achievement-oriented highly skilled late-matured players* or more than two operating factors below-average in a pattern (cluster 3-2), cannot be compensated at this point in time.

It is also interesting to see that it is not those clusters characterized by maximum scores in the in individual operating factors, such as cluster 3-1 with the highest net hope or cluster 3-4 with on average the most mature players, that have the highest probability of success. Instead, players with well-balanced profiles that mainly lie in the above-average range, appear to have the highest probability of success. This result agrees with the assertion by Reilly et al. (2000a, p. 669) – in this case confined to the field of physical fitness – that “players may not need to have an extraordinary capacity within any of the areas of physical performance but must possess a reasonably high level within all areas.”

The advantage of the person-oriented approach adopted in this study, as compared with the variable-oriented studies carried out before, is that it satisfies the theoretical and methodological demands of using *multidimensional* and *dynamic approaches* (Williams and Franks, 1998; Abbott and Collins, 2002; Abbott et al., 2005; Reilly et al., 2008; Vaeyens et al., 2008; Meylan et al., 2010; Unnithan et al., 2012; Phillips et al., 2014). This is demonstrated, among other things in the fact that it does not assume the same model for all players (for an overview, cf. Bergman and Andersson, 2010) but instead considers individual patterns and compensations effects. Using this approach, the object is not to find criteria for talent and deterministic connections between variables and specific outcomes, but instead to find talents and their probability of success. This precludes a direct comparison with the previously conducted studies and their classification rates (e.g., Falk et al., 2004; Huijgen et al., 2014) from a theoretical point of view.

### Limitations

The following critical issues must be taken into consideration as regards the study conducted. First, the holistic approach chosen has only been partially implemented by this study in looking at variables representing different dimensions. This procedure remains closer to the person as a whole than if one only studies individual subsystems such as motivation (Zuber et al., 2015) or training (Zibung and Conzelmann, 2013). On the other hand, when a holistic analysis is performed, individual variables have to represent an extensive range of phenomena, meaning that certain reservations must be expressed concerning the content validity of such variables. When scores are used, on the other hand, as for example in the field of technical skills and physical fitness, it should be critically noted that forming these contradicts the demand for a person-oriented approach in terms of renouncing the GLM (Bergman and Magnusson, 1997). Furthermore, for a truly holistic system of talented soccer players, the aspect of environmental conditions is still missing, which also play an important role (e.g., in the TID model of Abbott et al., 2005). In the present paper, only endogenous factors have been taken into account, which seems quite appropriate for a first attempt at approaching the developing athletes in a more holistic fashion than before.

Secondly, in interpreting the results it must be remembered that the promising young soccer talents analyzed constitute a highly selective sample and that the results should therefore only be considered in the context of the overall sample. Hence for example, the term “late developed” only applies with respect to other players within this sample, whereas one would expect all the players studied to be considered early developers compared with the average of all adolescents of this age (cf. Malina et al., 2005b; Meylan et al., 2010). However, since these results are hardly likely to be generalized to the general population, but primarily used on performance-oriented and thus pre-selected samples as part of the talent selection process, this problem would seem to be secondary. Nevertheless, strategies ought to be adopted much earlier on, in order to protect late-developing athletes from being placed at a disadvantage and from frequently not being selected as a result.

### Outlook

A sensible next step that would deal with the criticisms listed above, i.e., (a) the low representativeness of individual operating factors for the overall dimensions, and (b) the calculation of scores based on the GLM, would seem to be to extend the implementation of the person-oriented approach by using super-clusters. By serving as second-degree clusters, these would involve cluster allocations of different subsystems, e.g., motor behavior, personality and environment, as operating factors and thereby permit a distinctly broader implementation of the holistic concept. Furthermore, future longitudinal studies ought to check whether the identified clusters are also found in other sports and in other stages of development, and whether they are also associated with longer-term success in sports.

Despite the limitations mentioned, the results of this study indicate that the holistic patterns of talented soccer players in their youth can be a valid instrument for predicting the performance level at age 15.

## Author Contributions

CZ: substantial contributions to the conception or design of the work and the acquisition, analysis, and interpretation of data for the work; draft of the article; final approval of the version to be published; agrees to be accountable for all aspects of the work in ensuring that questions related to the accuracy or integrity of any part of the work are appropriately investigated and resolved. MZ: substantial contributions to the conception or design of the work and the acquisition, analysis, and interpretation of data for the work; critical revision of the drafts; final approval of the version to be published; agrees to be accountable for all aspects of the work in ensuring that questions related to the accuracy or integrity of any. AC: substantial contributions to the conception or design of the work and the acquisition and interpretation of data for the work; critical revision of the drafts; final approval of the version to be published; agrees to be accountable for all aspects of the work in ensuring that questions related to the accuracy or integrity of any part of.

## Conflict of Interest Statement

The authors declare that the research was conducted in the absence of any commercial or financial relationships that could be construed as a potential conflict of interest.

## Footnotes

1 For the analysis of reliability, the Spearman-Brown formula was used to estimate the reliability of the test at t_1_ (Eisinga et al., 2013). Due to the fact that only one attempt was performed in the yo-yo test and only the best attempt (out of five) was available for the countermovement jump from the Myotest accelerometric system, the reliability score of the sprint test could only be estimated.

2 Due to the fact that the players were told that the better of the two attempts would be counted, often resulting in a safe and a high-risk attempt, the reliability scores of the technical skills tests may be distorted.

3 Further information on the number of imputed cases and a comparison of the cluster solutions with and without imputed cases are available in the supplementary material.

4 Absolute transition rates are available in the supplementary material.
