# Supplementary material for: Holistic Patterns as an Instrument for Predicting the Performance of Promising Young Soccer Players – A 3-Years Longitudinal Study
Source: Front Psychol. 2016 Jul 27;7:1088. doi: 10.3389/fpsyg.2016.01088 (PMC4961718; doi:10.3389/fpsyg.2016.01088)
Supplement: Supplementary file 1 [file Data_Sheet_1.PDF]

Supplementary Material

Holistic patterns as an instrument for predicting the performance of promising young soccer players – a 3-year longitudinal study

Claudia Zuber[1]*, Marc Zibung[1] and Achim Conzelmann[1]

[1]Institute of Sport Science, University of Bern, Bern, Switzerland

*** Correspondence:**
Claudia Zuber
claudia.zuber@ispw.unibe.ch

1 Missing data

Since the proportion of the imputed cases at the first two measuring points is low (cf. Supplementary Table 1), we checked the cluster solution at the third measuring point, even though no differences were apparent in the distribution of the imputed cases across the four clusters ($X^2(3) = 2.13$; $p = .55$). The structural stability between the two cluster solutions is found to be relatively high (.11 < SS < .82), with the exception of the very small cluster (n=5), and the developmental types and antitypes also largely agree with expectations (Supplementary Figure 1). Seeing that the cluster analysis of the subsample (all players who were present for all three measuring points, n=59) must be viewed relatively critically, that no fundamental differences are apparent between the two cluster solutions, and that the power for determining developmental (anti-)types is also greater when the sample is larger, it seems justified to use the sample including imputed values.

Supplementary Table 1. Proportion of imputed cases in the cluster at the ages of 12, 13 and 14

**N=119**	**12 years**		**13 years**		**14 years**	
	n	%	*n*	%	*n*	%
Cluster 1	2	7.4	0	0	1	10.0
Cluster 2	4	15.4	4	11.1	15	37.5
Cluster 3	4	10.0	2	5.5	12	30.0
Cluster 4	5	19.2	1	3.4	10	34.5
**Total**	**15**	**12.6**	**7**	**5.6**	**38**	**31.9**

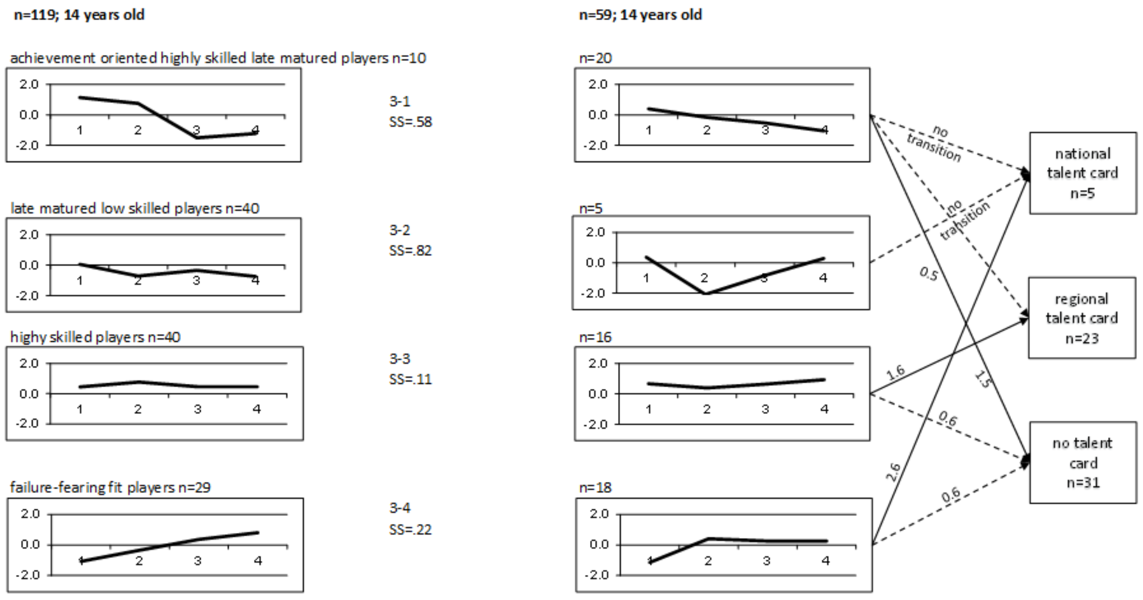

Supplementary Figure 1. Comparison (structural stability) of the cluster solutions with (n=119) and without (n=59) imputed cases.

2 Absolute transition rates

Supplementary Table 2. Absolute transition rates from age 12 to age 13

Origin-Cluster	(anti-)type	*n*	Target-Cluster
Cluster 1-1 n=27	——→	11	Cluster 2-1
		7	Cluster 2-2
		6	Cluster 2-3
		3	Cluster 2-4
Cluster 1-2 n=26		5	Cluster 2-1
	——→	17	Cluster 2-2
	- - -→	4	Cluster 2-3
	·······→	0	Cluster 2-4
Cluster 1-3 n=40	- - -→	3	Cluster 2-1
		10	Cluster 2-2
	——→	21	Cluster 2-3
		6	Cluster 2-4

Cluster 1-4 n=26		5	Cluster 2-1
	- - -→	2	Cluster 2-2
		5	Cluster 2-3
	⟶	14	Cluster 2-4

Supplementary Table 3. Absolute transition rates from age 13 to age 14

**Origin-Cluster**	**(anti-)type**	***n***	**Target-Cluster**
Cluster 2-1 n=24		1	Cluster 3-1
	⟶	12	Cluster 3-2
	- - -→	4	Cluster 3-3
		7	Cluster 3-4
Cluster 2-2 n=36		5	Cluster 3-1
	⟶	19	Cluster 3-2
	- - -→	5	Cluster 3-3
		7	Cluster 3-4
Cluster 2-3 n=36		4	Cluster 3-1
	- - -→	6	Cluster 3-2
	⟶	23	Cluster 3-3
	- - -→	3	Cluster 3-4
Cluster 2-4 n=23	·········→	0	Cluster 3-1
	- - -→	3	Cluster 3-2
		8	Cluster 3-3
	⟶	12	Cluster 3-4

Supplementary Table 4. Absolute transition rates from age 14 to performance criterion

**Origin-Cluster**	**(anti-)type**	***n***	**Target-Cluster**
Cluster 3-1 n=10	·········→	0	national talent card (n=12)
		2	regional talent card (n=39)
		8	no talent card (n=68)
Cluster 3-2 n=40	·········→	0	national talent card (n=12)
	- - -→	7	regional talent card (n=39)
	⟶	33	no talent card (n=68)
Cluster 3-3 n=40	⟶	9	national talent card (n=12)
		15	regional talent card (n=39)
	- - -→	16	no talent card (n=68)
Cluster 3-4 n=29		3	national talent card (n=12)
	⟶	15	regional talent card (n=39)
	- - -→	11	no talent card (n=68)
